# Effectiveness of Multi‐Modal Blood Management in Bernese Periacetabular Osteotomy and Periacetabular Osteotomy with Proximal Femoral Osteotomy

**DOI:** 10.1111/os.12794

**Published:** 2020-10-11

**Authors:** Ji‐jun Shang, Zhen‐dong Zhang, Dian‐zhong Luo, Hui Cheng, Hong Zhang

**Affiliations:** ^1^ Department of Orthopaedics Orthopaedic Hospital of Xinmi City Zhengzhou China; ^2^ Department of Orthopaedics The Fourth Medical Center of Chinese People's Liberation Army General Hospital Beijing China; ^3^ Department of Orthopaedics The Seventh Medical Center of Chinese People's Liberation Army General Hospital Beijing China

**Keywords:** Blood preservation, Blood transfusion, Congenital, Hip dislocation, Intraoperative period, Osteotomy, Postoperative hemorrhage

## Abstract

**Objective:**

Bernese periacetabular osteotomy (PAO), an effective treatment for patients with developmental dysplasia of the hip (DDH), is characterized by wide exposure, cancellous bone surgery, and difficult techniques. In addition, the hip joint is deep and of rich muscles and neurovascular supply, which significantly increases bleeding. For patients who had combined proximal femoral osteotomy (PFO), the blood loss may be tremendous. The blood management for PAO is still challenging. We aimed to evaluate the effectiveness of multi‐modal blood management for PAO and PAO combined with PFO.

**Patients and Methods:**

We retrospectively evaluated patients who had PAO with or without combined procedures from June 2010 to December 2018 in our department. The multi‐modal blood management protocol included three parts: (i) pre‐operation – autologous component blood donation and iron supplement/erythropoietin; (ii) during operation – controlled hypotension anesthesia, intraoperative auto‐blood transfusion, tranexamic acid (20 mg/kg, IV / 0.5 g local), and standardized surgical procedure to shorten surgical time; and (iii) post‐operation – no drainage used, selective allo‐blood transfusion, and ice packing technique. As the lacking of the above standard blood management protocol during PAO or PAO + PFO initially, we divided all the patients into three groups: Group A (PAO) – before protocol started, 74 hips; Group B (PAO) – after protocol finalized, 178 hips; Group C (PAO + PFO) – after protocol finalized, 55 hips. The intraoperative blood loss, surgical time, allo‐transfusion rate, pre‐ and postoperative hemoglobin were compared among groups.

**Results:**

Both the general characteristics and preoperative hemoglobin were comparable among the three groups (*P* < 0.001). The intraoperative blood loss was 797.1 ± 312.2, 381.7 ± 144.0 and 544.1 ± 249.1 mL, respectively. The surgical time was 109.6 ± 18.5, 80.2 ± 20.0 and 154.3 ± 44.7 min, respectively. The allo‐transfusion rate was 86.5%, 0%, and 2%, respectively. The mean decreased value of hemoglobin on the first postoperative day of group B and group C was greater than that of group A, which was associated with the higher allo‐transfusion rate of group A. However, on the third postoperative day, the mean decreased value of hemoglobin of group B was less than that of group A and group C.

**Conclusion:**

Perioperative multi‐modal blood management for PAO or PAO + PFO can significantly decrease intraoperative blood loss, reduce allo‐transfusion rate from over 80% to 0%, and ensure the rapid recovery of postoperative hemoglobin level.

## Graphical abstract

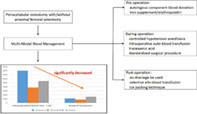

Perioperative multi‐modal blood management for periacetabular osteotomy with/without proximal femoral osteotomy can significantly decrease intraoperative blood loss, reduce allo‐transfusion rate from over 80% to 0%, and ensure the rapid recovery of postoperative hemoglobin level.

## Introduction

Developmental dysplasia of the hip (DDH) is one of the common hip deformities in China. Bernese periacetabular osteotomy (PAO) has now become an effective and the most popular surgical treatment for the management of DDH^1^, ^2^, ^3^. However, PAO, a technically demanding surgery, is characterized by extensive exposure, cancellous osteotomy around acetabulum, and difficult techniques. Besides, the hip joint is deep and the acetabulum is of rich vascularity, which significantly increases the volume of bleeding^4^. In addition, for patients with femoral abnormality, such as abnormal femoral torsion angle, femoral valgus or varus abnormality, the proximal femoral osteotomy (PFO) is also needed^5^, ^6^, thus leading to the potential for substantial blood loss. Previous studies showed that the intraoperative blood loss of PAO ranged from 300 mL to 4500 mL^2^, ^3^, ^4^. The acute anemia caused by tremendous perioperative blood loss can postpone the postoperative rehabilitation and prolong the length of hospital stay, which is against the theory of enhanced recovery after surgery (ERAS)^7^. The subsequent allogenic blood transfusion may increase the risk of transfusion‐associated morbidity, such as anaphylaxis, acute lung injury, and transfusion‐transmitted infection^8^, ^9^, ^10^. Therefore, methods to control bleeding and reduce allogenic blood transfusion are critical.

Several methods, including preoperative autologous blood donation, intraoperative use of tranexamic acid, and intraoperative auto‐blood transfusion, have been reported for the management of blood loss of PAO^4^, ^11^. However, the independent strategy can not significantly decrease the allogenic blood transfusion rate. Combining all useful methods and adding some new strategies may be helpful. Currently, there is still lack of recommended protocol or successful combination of different strategies for blood management in PAO with/without PFO.

Taking into account all kinds of strategies and our clinical experience regarding the blood management of PAO ± PFO, the integrated perioperative multi‐modal blood management for PAO or PAO + PFO was finalized in 2015 in our department. The multi‐modal blood management protocol included three parts: (i) pre‐operation – autologous component blood donation and iron supplement/erythropoietin; (ii) intra‐operation – controlled hypotension anesthesia, intraoperative auto‐blood transfusion, tranexamic acid (20 mg/kg, IV / 0.5 g local)^12^, ^13^, ^14^, and standardized surgical procedure to shorten surgical time; and (iii) post‐operation – no drainage used, selective allo‐blood transfusion and ice packing technique. According to the time periods, we divided the patients into three groups, PAO before protocol finalized, PAO after protocol finalized, and PAO + PFO after protocol finalized. The aims of this study were: (i) to illustrate our perioperative blood management strategies of PAO and PAO + PFO briefly; (ii) to identify the allo‐transfusion rate of patients with the multi‐modal blood management; and (iii) to evaluate the effectiveness of multi‐modal blood management for PAO and PAO + PFO.

## Patients and Methods

The PAO procedure has been carried out in our department since 2010. The detailed surgical techniques of this procedure have been described elsewhere^1^, ^2^, ^3^. As we were lacking an integrated multi‐modal blood management protocol initially, we only used the controlled hypotension anesthesia^15^, ^16^, ^17^, ^18^ and intraoperative blood collection and re‐infusion^4^ strategy for suitable patients. Intraoperatively, under the general anesthesia, the controlled hypotension anesthesia was adopted to maintain systolic pressure between 85 mm Hg and 95 mm Hg, diastolic pressure between 40 mm Hg and 70 mm Hg, and the mean arterial pressure between 60 mm Hg and 65 mm Hg at the time of osteotomy. The intraoperative auto‐transfusion system was used to return the washed red blood cells to the patient. We choose patients consecutively at the initial period (from June 2010 to June 2011) who met the following criteria as Group A (before protocol started): an age of 13–50 years with DDH, unilateral and primary surgery, no preoperative anemia or hematological diseases, normal coagulation function. Patients with combined surgery, including surgical hip dislocation procedure or hip arthroscopy were excluded. As most of the DDH patients were female, we also exclude the male patients in order to eliminate the bias between genders.

Apart from controlled hypotension anesthesia and intraoperative blood collection and re‐infusion, a series of strategies have been involved in blood management for PAO procedure gradually in our department. Preoperatively, we conducted the autologous component blood donation. The indications for preoperative autologous donation were as follows: aged >12 years old, body mass index was normal, hemoglobin >110 g/L, and hematocrit >34%. The time of blood donation was at least 48 h before surgery and the donation volume was less than 12% of the whole body blood volume. The iron sucrose (200 mg, IV) or erythropoietin (PRN) was routinely used after blood donation. Tranexamic acid was used by combining intravenous (20 mg/kg) and local (0.5 g, just before incision closure) methods^19^, ^20^. As for the surgical techniques, we emphasized the importance of personalized surgical plan, complete hemostasis, accurate and fast osteotomy, and acetabular rotation^21^. The above standardized surgical procedure played an important role in shortening surgical time. No drainage was used and the ice‐packing cooling technique was used on incision postoperatively. In patients with hemoglobin <7.0 g/dL or hemoglobin <9.0 g/dL yet with symptoms related with anemia, chest pain caused by myocardial ischemia, mental disturbance, oliguria, tachycardia, or hypotension postoperatively, the allogenic blood transfusion should be applied^22^, ^23^.

The above blood management strategies were gradually applied in our clinical practice. The integrated perioperative multi‐modal blood management protocol was developed in January 2015. Therefore, we enrolled patients who received the whole protocol as well as met the same inclusion and exclusion criteria as Group A from January 2015 to December 2018, Group B (PAO, after protocol finalized), and Group C (PAO + PFO, after protocol finalized).

We retrospectively reviewed the clinical data of all patients in the three groups. The general characteristics including age, weight, height, and body mass index and the intraoperative blood loss, surgical time, allo‐transfusion volume and rate, pre‐ and postoperative hemoglobin were compared among groups. Postoperative hemoglobin was collected every other day during the hospital stay (postoperative days 1, 3, and 5).

### *Statistical Analysis*

SPSS version‐25.0 statistical software (IBM) was used to perform the statistical analysis. Continuous variables were reported as the mean and standard deviation (SD) and categorical variables were reported as percentages. The ANOVA variance analysis was used to compare continuous variables among different groups. *P* < 0.05 was considered to be significant.

## Results

### *General Characteristics*

There were 74 cases in group A, 178 cases in group B, and 55 cases in group C. The height, weight, body mass index, and preoperative hemoglobin were comparable among the three groups (Table 1). Patients in group C were younger than those in groups A and B.

**TABLE 1:** Comparison of general characteristics and preoperative hemoglobin among 3 groups

Characteristics	Group A	Group B	Group C	*P* value
No. of cases	74	178	55	‐
Age (years)	28.5 ± 7.6	31.1 ± 7.9	21.3 ± 6.2	<0.001
Height (cm)	160.5 ± 5.9	158.4 ± 21.3	160.2 ± 15.5	0.615
Weight (kg)	56.3 ± 8.9	57.3 ± 8.1	57.1 ± 17.2	0.787
Body mass index	21.9 ± 3.4	22.1 ± 2.8	21.1 ± 3.3	0.069
Hemoglobin (g/L)	125.8 ± 10.5	128.8 ± 10.1	129.1 ± 9.9	0.081

The values are given as the mean and standard deviation.

### *Intraoperative Blood Loss, Surgical Time, and Transfusion Rate*

**Fig. 1:**
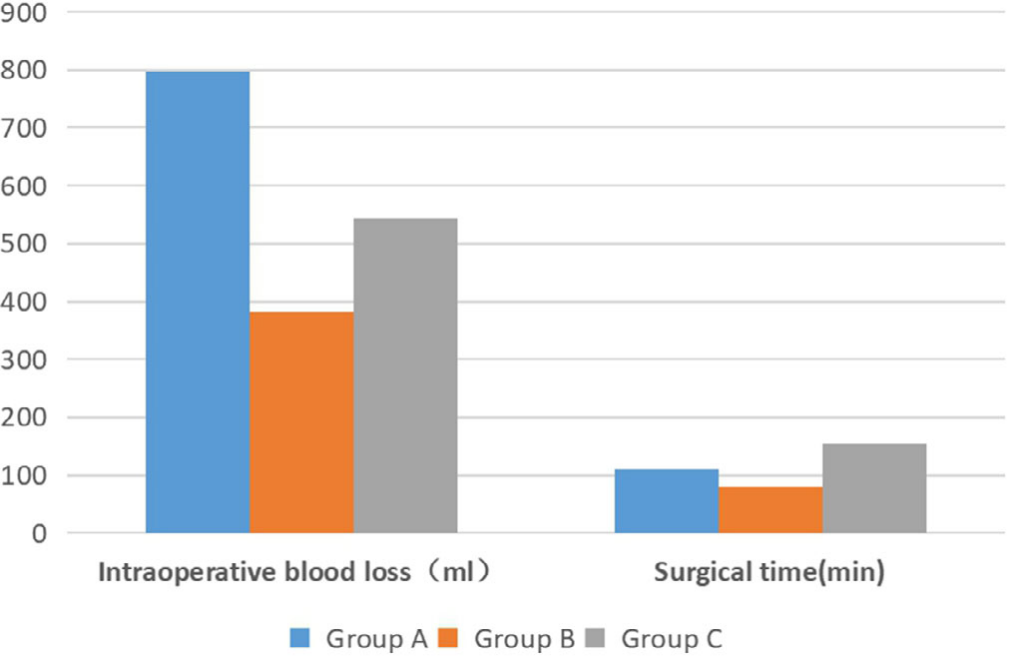
The intraoperative blood loss and surgical time of three groups.

The intraoperative blood loss was 797.1 ± 312.2 mL, 381.7 ± 144.0 mL, and 544.1 ± 249.1 mL, respectively (*P* < 0.001). The surgical time was 109.6 ± 18.5 min, 80.2 ± 20.0 min, and 154.3 ± 44.7 min, respectively (*P* < 0.001) (Fig. 1). The allo‐transfusion rate was 86.5%, 0%, and 2%, respectively (*P* < 0.001). There were 64 patients who required allogenic transfusion in group A (average 401.1 mL per patient). No patients in group B and only one patient in group C required allogenic transfusion.

### *Value of Hemoglobin Change*

The mean decreased value of hemoglobin on the first postoperative day of group B and C was greater than that of group A, which was associated with the higher allo‐transfusion rate of group A. However, on postoperative day 3, the mean decreased value of hemoglobin of group B was lesser than that of group A and C. On postoperative day 5, the decreased value of hemoglobin of group A and B was 32.0 ± 14.5 and 32.0 ± 11.0 mL, respectively, and the difference was not significant. Besides, compared with day 3, the level of hemoglobin on day 5 was significantly higher in each group. The details of the above results are shown in Table 2.

**TABLE 2:** The hemoglobin level and the decrease of hemoglobin on postoperative days

Hemoglobin	Group A	Group B	Group C	*P* value
Hb‐Day 1 (g/L)	100.5 ± 13.7	100.5 ± 10.6	93.6 ± 10.1	<0.001
Decrease of Hb‐Day 1 (g/L)	25.3 ± 13.0	28.3 ± 10.0	35.4 ± 12.5	<0.001
Hb‐Day 3 (g/L)	89.5 ± 16.2	94.3 ± 10.7	85.2 ± 12.6	<0.001
Decrease of Hb‐Day 3 (g/L)	36.3 ± 17.0	34.3 ± 9.6	43.2 ± 14.6	0.004
Hb‐Day 5 (g/L)	93.9 ± 13.2	97.0 ± 10.0	87.4 ± 11.2	<0.001
Decrease of Hb‐Day 5 (g/L)	32.0 ± 14.5	32.0 ± 11.0	42.1 ± 13.0	<0.001

The values are given as the mean and standard deviation. Hb, hemoglobin.

## Discussion

PAO is currently the most effective procedure for treating patients with symptomatic DDH. It was reported that PAO is a complex procedure with substantial blood loss and high allogenic blood transfusion rate^1^, ^2^, ^3^, ^4^. Thus, it poses a great importance on blood‐conserving strategies. Previous studies about blood management mainly focus on hip or knee arthroplasty. However, compared with arthroplasty, the blood loss of PAO was higher and the blood management was more challenging. There is still no certain guideline on blood management of PAO, as limited studies have analyzed all useful strategies with small sample size^4^, ^11^, ^19^, ^20^, ^24^. The current study described an integrated perioperative multi‐modal blood management for PAO with/without PFO. Our findings clearly demonstrate the advantages and efficacy of this multi‐modal blood management for PAO or PAO + PFO. By using these strategies we were able to reduce intraoperative blood loss and allo‐transfusion rate significantly.

Previous studies have reported several strategies in reducing allo‐transfusion rate or blood loss during PAO. Atwal *et al*.^4^ reported that preoperative autologous blood donation was a safe and cost‐effective method of blood management. Wassilew *et al*.^20^ showed a significant reduction in blood loss and transfusion rate in patients with tranexamic acid (10 mg/kg/h). A recent meta‐analysis also verified the efficacy of intravenous anti‐fibrinolytic agents in reducing blood loss during PAO^19^. However, the patients' blood loss was still tremendous even though they received tranexamic acid. According to a study by Wassilew *et al*.^20^, the mean blood loss in patients with tranexamic was 1500 mL. In our study, the mean blood loss of group B was 381.7 mL, which was lower than all previous reports. Therefore, all useful methods for blood management should be taken into consideration. The allo‐transfusion rate of group A is 86.5% in our study (average 401.1 mL per patient). With the utilization of multi‐modal blood management, no patient needs allogenic blood transfusion (group B), thus achieving blood conservation.

Both Lee *et al*.^11^ and Novais *et al*.^24^ concluded that the longer operative time was associated with increased blood loss. Lee *et al*. reported average surgical duration as 4.25 h. They found that blood loss increased by 11.1% for every 1 h increase in surgical duration. Novais *et al*. reported the mean operative time of PAO was 273 min, and every 10 min increase would cause 1.1% increase in blood loss. In the current study, the initial mean operative time was 109.6 min. After a series of measures have been taken to shorten operative time, including personalized surgical plan, accurate and fast osteotomy, and acetabular rotation, we can now finish one PAO within 80.2 ± 20.0 min.

For patients with concomitant femoral deformities, the proximal femoral rotation osteotomy or varus/valgus osteotomy should be performed on young patients^5^, ^6^. Undoubtedly, due to the prolonged surgical duration and additional incision, the additional procedure is associated with increased bleeding during surgery. We analyzed the data of patients who received PAO combined with PFO (group C) and found that the blood loss was greater than patients with PAO alone (544.1 ± 249.1 *vs* 381.7 ± 144.0 mL). A univariate analysis demonstrated that additional procedures (osteochondroplasty, labral repair, anterior hip arthrotomy) were associated with longer operative time and more bleeding^24^. To our knowledge, our study is the first to investigate the blood management on PAO + PFO. The results showed that patients with PAO + PFO had longer surgical time (154.3 ± 44.7 *vs* 109.6 ± 18.5 min) but less blood loss (544.1 ± 249.1 *vs* 797.1 ± 312.2 mL) than patients who had PAO without multi‐modal blood management. Furthermore, though there is great trauma during PAO + PFO, the allogenic blood transfusion rate is extraordinarily low (2%). Therefore, PAO with concomitant procedures can also benefit from the multi‐modal blood management.

We found the drop in hemoglobin on the first postoperative day of patients who had multi‐modal blood management was greater than that of patients without multi‐modal blood management. The possible explanation was that most patients in group A had allogenic blood transfusion. However, on postoperative day 3, the drop in hemoglobin of group B patients was lesser than that of group A (34.3 ± 9.6 *vs* 36.3 ± 17.0 g/L). The mean hemoglobin level of group B, though with a 0% allogenic transfusion rate, was higher than that of group A (94.3 ± 10.7 *vs* 89.5 ± 16.2 g/L). The hemoglobin level began to rise from postoperative day 5, when the drop in hemoglobin was comparable between group A and B. So, it is evident that the multi‐modal blood management can ensure the rapid recovery of hemoglobin level without additional allogenic transfusion.

Our study has some limitations. First, it is a retrospective study from one single institution. A multi‐center prospective study may be more persuasive. Second, the patients were grouped according to time period, which may result in bias from different times. However, the preoperative general characteristics were comparable among the three groups. Third, the current study mainly focused on the pre‐ and 5 days of postoperative hemoglobin, yet the follow‐up data was also important in evaluating patients' recovery.

In conclusion, by utilizing perioperative multi‐modal blood management, PAO can be finished within 80 ± 20.0 min without massive intraoperative blood loss. Even for patients with combined PFO, their postoperative hemoglobin level can also recover rapidly without additional allogenic transfusion. The perioperative multi‐modal blood management can reduce allo‐transfusion rate from over 80% to 0%.

## Conflict of Interest

The authors declare that they have no conflict of interest.
